# Beyond weight loss: Effect of GLP-1 receptor agonist therapy on the urological health

**DOI:** 10.1016/j.eucr.2026.103368

**Published:** 2026-02-09

**Authors:** Arisha Tariq, Adrian Garza Gangemi, Jaime Herrera Caceres

**Affiliations:** Department of Urology, Penn State Health, Hershey, PA, USA

## Abstract

**Background:**

Glucagon-like-peptide-1 receptor agonists (GLP-1-RA) are increasingly prescribed medications with well-documented gastrointestinal adverse effects. Urologic adverse effects remain poorly characterized.

**Case presentation:**

A 40-year-old male developed reproducible urinary frequency, penile pruritus, dysuria, pelvic/perineal pain, and sexual dysfunction 3-4 weeks after initiating tirzepatide therapy. Comprehensive infectious evaluation remained negative. Symptoms improved 4 days after discontinuing tirzepatide with complete resolution in 10 days and no recurrence two weeks after last injection.

**Conclusion:**

This case describes novel urologic adverse effects associated with GLP-1-RA therapy. Clinicians should consider medication-related etiologies in patients with atypical urogenital symptoms with negative infectious workup during GLP-1-RA use.

## 1 Introduction

Glucagon-like peptide-1 (GLP-1) receptor agonists have become a popular modern therapy for type 2 diabetes and obesity, exerting their effects through enhancing glucose dependent insulin secretion, delayed gastric emptying, and central appetite suppression.^1^ In the U.S, there is over 6.9 million adults reported to be using injectable GLP-1 receptor agonists in 2024 with a projected increase of millions of patients per year while overall spending on these medications has rapidly increased by more than 500% from 2018 to 2023.^2^^,^^3^

Among these agents, tirzepatide is a dual glucose-dependent insulinotropic polypeptide (GIP) and GLP-1 receptor agonist that has demonstrated efficacy in both glycemic control and weight loss with a safety profile that is broadly similar to other GLP-1 receptor agonists.^4^^,^^5^ Commonly reported side effects of GLP-1 receptor agonists and tirzepatide include gastrointestinal symptoms such as nausea, vomiting, diarrhea, and constipation most of which are typically dose dependent and often mitigate over time with careful monitoring.^4^^,^^6^

Despite reported knowledge of gastrointestinal and metabolic effects, there is limited evaluation of urogenital or lower urinary tract symptoms associated with GLP-1 receptor agonist therapy in recent studies. Randomized controlled trials and meta-analyses of GLP-1 receptor agonists including tirzepatide have not consistently identified the rates of urinary tract infections or other genitourinary adverse events as significant compared with control groups.^7^^,^^8^ Recent pooled analyses indicate that tirzepatide does not increase the risk of urinary tract infection, nephrolithiasis, or other renal adverse outcomes compared to placebo or other diabetes therapies.^9^ Importantly, available GLP-1 receptor agonist clinical trial data and FDA prescribing information does not list urinary frequency, dysuria, or prostatitis-like symptoms as established adverse effects.^10^

Infection risks associated with GLP-1 receptor agonists remain a point of investigation. Trials with exenatide and other incretin-based agents have reported urinary tract infections among common adverse events though causal relationships were not established and physiologic explanations have not yet been determined.^1^ Comparative safety analyses have suggested that differences in adverse event profiles between specific GLP-1 agents including evidence of urogenital symptoms remains limited.^5^

The scarcity of data addressing unusual urogenital adverse reactions highlights a critical gap in the current literature particularly given the rapid expansion of GLP-1 receptor agonist use for weight management. Case reports and pharmacological studies have played an important role in identifying rare or idiosyncratic reactions for other drug classes and may be similarly valuable for GLP-1 receptor agonists. Here we present a case of a novel prostatitis-like urologic adverse effect temporally associated with tirzepatide therapy that resolved after medication discontinuation, thereby expanding the emerging safety profile of this widely used class of medications.

## 2 Case Presentation

A 40-year-old male with a past medical history significant for hypertension, lumbar disc herniation, and a prior cholecystectomy presented with a subacute onset of persistent urogenital symptoms subsequently found to be associated his initiation of a glucagon-like peptide-1 (GLP-1) receptor agonist. Tirzepatide had been initiated through an online medical service at a dose of 2.5 mg weekly. He had no prior history of lower urinary tract symptoms (LUTS), urinary tract infections, sexually transmitted infections, chronic pelvic pain, neuropathy, or urologic disease. He also reported no new products, changes in routine, sexual partners, and no history of autoimmune conditions.

Approximately three to four weeks after starting tirzepatide 2.5 mg, patient developed urinary frequency and a sensation of pressure beginning in the posterior urethra and extending to the tip of the penis. These symptoms were accompanied by pronounced penile pruritus described as internal without visible rash, erythema, or skin breakdown. He also reported burning and pain with urination, painful erections, inability to ejaculate, and pain during sexual activity. He endorsed perineal, anal, and urethral pain but denied having any testicular or scrotal pain. He denied concerning symptoms of hematuria, fever, chills, gastrointestinal symptoms, or other acute systemic complaints.

At approximately four weeks after his initial dose, patient visited an urgent care and was empirically treated for a urinary tract infection with a seven-day course of ciprofloxacin with unremarkable urinalysis testing. Due to persistent symptoms, he was then subsequently treated with nitrofurantoin 100 mg and later with trimethoprim-sulfamethoxazole without any improvement. During weeks five to six of therapy, he had diminished efficacy of the GLP-1 medication and received a higher dose of tirzepatide injection at 5mg. His symptoms continued and he subsequently returned to urgent care where he was prescribed an additional course of treatment reported to include fluconazole and an over the counter topical antifungal cream. His urinalysis results remained unremarkable, and trial of topical therapy emphasized that the pruritus remained internal in nature. Urinalysis, urine culture, sexually transmitted infection testing, and yeast culture were all negative.

Following persistent symptoms, he was advised to pursue urologic evaluation. He reported that his urogenital symptoms consistently worsened within 24 to 48 hours following each weekly injection and partially improved prior to the subsequent dose. After the third injection at 5mg dose of tirzepatide he presented to a primary care wellness center where infection was ruled out, he was diagnosed with acute prostatitis and treated with doxycycline without clinical improvement.

By approximately six weeks after symptom onset, patient had received a total of seven weeks of tirzepatide therapy. He noted further exacerbation of symptoms following the third 5 mg injection and subsequently discontinued tirzepatide therapy. Four days after stopping the medication, his symptoms began to improve, and patient had complete resolution gradually over a total of approximately ten days following discontinuation. Over two weeks after his final tirzepatide injection the patient reported complete resolution of urinary, genital, and sexual symptoms. He has not restarted tirzepatide or any other GLP-1 receptor agonist and has experienced no recurrence of symptoms.

## 3 Discussion

This case describes a potential novel urologic adverse effect temporally associated with GLP-1 receptor agonist therapy, characterized by urinary frequency, penile pruritus, dysuria, pelvic and perineal pain, and sexual dysfunction in the absence of identifiable infection. This syndrome is nonspecific and may mimic chronic pelvic pain syndrome in men. The reproducible temporal relationship between weekly tirzepatide administration and symptom exacerbation followed by resolution after drug discontinuation supports a medication-related adverse effect rather than an infectious or structural urologic etiology. This case expands our current knowledge of the safety profile of GLP-1 receptor agonists which are largely focused on gastrointestinal, metabolic, and endocrine adverse events.^1^^,^^4^, ^5^, ^6^

Large randomized control trials and meta-analyses evaluating GLP-1 receptor agonists such as tirzepatide and semaglutide have not yet demonstrated an increased risk of urinary tract infections or other genitourinary adverse outcomes when compared to placebo or other standard therapies.^7^, ^8^, ^9^ Regulatory labeling and safety summaries also do not include any urinary frequency, dysuria, pelvic pain, or prostatitis-like symptoms as recognized adverse effects of these medications.^10^

Observational studies exploring the urinary outcomes in selected populations have suggested either neutral or potentially protective effects of GLP-1 receptor agonists on lower urinary tract symptoms, further demonstrating the unusual nature of this patient's presentation.^11^^,^^12^ In a retrospective cohort of non-diabetic adults with overactive bladder treated with onabotulinumtoxin-A, concomitant GLP-1 receptor agonist use was associated with lower rates of urinary retention and urinary tract infections while other studies have reported preliminary associations with changes in urinary peptidomes or symptom patterns without established causation.^11^^,^^12^

The absence of objective evidence of infection in this case is particularly notable. Despite empiric antibiotic and antifungal therapy with fluoroquinolones, nitrofurantoin, trimethoprim-sulfamethoxazole, fluconazole, and doxycycline his symptoms persisted without significant improvement. Infectious sources had been repeatedly excluded through negative urinalysis, urine cultures, sexually transmitted infection testing, and yeast cultures. These findings argue against acute or chronic bacterial prostatitis and highlight the risk of diagnostic anchoring when persistent LUTS, pelvic or urethral symptoms are presumed to be infectious in origin, a phenomenon well described in the chronic prostatitis/chronic pelvic pain syndrome literature.^13^

Several potential mechanisms may help explain the observed symptoms. GLP-1 receptors are expressed beyond the gastrointestinal tract, in autonomic nervous system pathways involved in visceral sensation and pelvic organ function.^14^ Experimental and clinical data suggest that GLP-1 receptor agonists may influence autonomic tone, sensory processing, and muscle activity.^15^ Neuropathic pruritus and dysesthesia particularly when described as internal and without visible dermatologic findings are increasingly recognized as manifestations of altered sensory signaling rather than cutaneous disease.^16^ The patient's reproducible worsening of symptoms within 24 to 48 hours of each injection, followed by partial improvement before the next dose, further supports a pharmacodynamic effect rather than coincidental symptom fluctuation.

Post-marketing pharmacological studies have begun to identify unexpected adverse events associated with tirzepatide and other GLP-1 receptor agonists that were not prominent in pre-approval trials. Recent analysis of the FDA Adverse Event Reporting System has identified a broad spectrum of non-gastrointestinal adverse events, including neurologic and sensory complaints, though causality cannot be established from these data alone.^17^^,^^18^ Case reports such as this play an important role in studying such adverse effects and generating the basis for future investigation.

As GLP-1 receptor agonists are increasingly prescribed for weight loss, the recognition of any atypical and non-metabolic adverse effects becomes increasingly important. Chronic pelvic pain syndrome in both men and women is clinically challenging to diagnose and treat. Due to the dramatic global increase in the use of this medication, identifying these symptoms during concurrent use is becoming increasingly relevant. Identifying medication-related urologic symptoms early may help prevent unnecessary antimicrobial exposure, diagnostic delays, and ultimately patient morbidity. This case underscores the importance of considering medication effects in the differential diagnosis of prostatitis-like syndromes with negative infectious workup, particularly in patients receiving GLP-1 receptor agonist therapy.

## 4 Conclusion

This case highlights a urologic syndrome that may mimic chronic pelvic pain syndrome/chronic prostatitis. This adverse effect that has not previously been reported to be associated with the use of GLP-1 receptor agonist therapy which resolved following medication discontinuation. When patients present with persistent urogenital symptoms despite a negative infectious evaluation, clinicians should consider GLP-1 receptor agonists as a potential contributing factor. As use of these medications continues to grow, further investigation is warranted to better understand the prevalence, underlying physiologic mechanisms, and the full range of associated clinical presentations.

## CRediT authorship contribution statement

**Arisha Tariq:** Conceptualization, Data curation, Investigation, Writing – original draft, Writing – review & editing. **Adrian Garza Gangemi:** Supervision, Writing – review & editing. **Jaime Herrera Caceres:** Conceptualization, Supervision, Writing – review & editing.

## Ethics and consent

Informed consent was obtained from the patient for publication of this case report. At our institution, ethical approval is not required for de-identified case reports.

## Source of funding

None.

## Conflicts of interest

None.

## References

[1] Filippatos T.D., Panagiotopoulou T.V., Elisaf M.S. (2014). Adverse effects of GLP-1 receptor agonists. Rev Diabet Stud : Reg Dev Stud.

[2] Centers for Disease Control and Prevention, National Center for Health Statistics (2025). https://www.cdc.gov/nchs/products/databriefs/db537.htm.

[3] Tsipas S., Dieleman J.L., Shah N.D., Soni A., Roehrig C. (2025). Spending on glucagon-like peptide-1 receptor agonists among U.S. adults. JAMA Netw Open.

[4] Mishra R., Raj R., Elshimy G. (2023). Adverse events related to tirzepatide. J Endocr Soc.

[5] Xie Z., Liang Z., Xie Y., Zheng G., Cao W. (2025). Comparative safety of GLP-1/GIP Co-Agonists versus GLP-1 receptor agonists for weight loss in patients with obesity or overweight: a systematic review. Diabetes Metab Syndr Obes.

[6] Salvador R., Abreu C., Monteiro M.P. (2025). Semaglutide as a GLP-1 agonist: efficacy and adverse effects. Pharmaceuticals.

[7] Kamrul-Hasan A., Patra S., Dutta D. (2025). Renal effects and safety of tirzepatide in subjects with and without diabetes: a systematic review and meta-analysis. World J Diabetes.

[8] Ljungberg C., Thomsen R.W., Knop F.K. (2025). Risk of urogenital infections in people with type 2 diabetes initiating GLP-1 receptor agonists versus SGLT2 inhibitors: a nationwide cohort study. Diabetes Care.

[9] Hammad M.A.M., Abdelrahman M.M., Elsamadicy A.A. (2025). Concurrent GLP-1 receptor agonist use is associated with reduced urinary adverse events following onabotulinumtoxinA treatment in non-diabetic adults with overactive bladder. Toxins.

[10] U.S. Food and Drug Administration (2023). Ozempic (semaglutide) prescribing information. https://www.accessdata.fda.gov/drugsatfda_docs/label/2023/209637s020s021lbl.pdf.

[11] Sandler M.D., Patel H.D., Helfand B.T. (2025). Effects of GLP-1 receptor agonists on overactive bladder symptom patterns. Urol Pract.

[12] Lohia S., Kumar V., Sharma A. (2023). Effect of treatment with GLP-1 receptor agonists on the urinary peptidome of patients with type 2 diabetes mellitus. medRxiv.

[13] Pontari M.A., Ruggieri M.R. (2008). Mechanisms in prostatitis/chronic pelvic pain syndrome. J Urol.

[14] Holst J.J., Rosenkilde M.M. (2020). GIP as a therapeutic target in diabetes and obesity: insight from incretin Co-agonists. J Clin Endocrinol Metabol.

[15] Drucker D.J. (2018). Mechanisms of action and therapeutic application of glucagon-like Peptide-1. Cell Metab.

[16] Steinhoff M., Oaklander A.L., Szabó I.L., Ständer S., Schmelz M. (2019). Neuropathic itch. Pain.

[17] Ou Y., Cui Z., Lou S. (2024). Analysis of tirzepatide in the US FDA adverse event reporting system (FAERS): a focus on overall patient population and sex-specific subgroups. Front Pharmacol.

[18] Liu L. (2024). A real-world data analysis of tirzepatide in the FDA adverse event reporting system (FAERS) database. Front Pharmacol.

